# Advances in Medical Revascularisation Treatments in Acute Ischemic Stroke

**DOI:** 10.1155/2014/714218

**Published:** 2014-12-30

**Authors:** H. Asadi, B. Yan, R. Dowling, S. Wong, P. Mitchell

**Affiliations:** ^1^Melbourne Brain Centre, Department of Medicine, Royal Melbourne Hospital, University of Melbourne, Parkville, VIC, Australia; ^2^Radiology Department, Western Hospital, Footscray, VIC, Australia

## Abstract

Urgent reperfusion of the ischaemic brain is the aim of stroke treatment and there has been ongoing research to find a drug that can promote vessel recanalisation more completely and with less side effects. In this review article, the major studies which have validated the use and safety of tPA are discussed. The safety and efficacy of other thrombolytic and anticoagulative agents such as tenecteplase, desmoteplase, ancrod, tirofiban, abciximab, eptifibatide, and argatroban are also reviewed. Tenecteplase and desmoteplase are both plasminogen activators with higher fibrin affinity and longer half-life compared to alteplase. They have shown greater reperfusion rates and improved functional outcomes in preliminary studies. Argatroban is a direct thrombin inhibitor used as an adjunct to intravenous tPA and showed higher rates of complete recanalisation in the ARTTS study with further studies which are now ongoing. Adjuvant thrombolysis techniques using transcranial ultrasound are also being investigated and have shown higher rates of complete recanalisation, for example, in the CLOTBUST study. Overall, development in medical therapies for stroke is important due to the ease of administration compared to endovascular treatments, and the new treatments such as tenecteplase, desmoteplase, and adjuvant sonothrombolysis are showing promising results and await further large-scale clinical trials.

## 1. Introduction

Stroke is a major public health problem worldwide and is considered the third most costly health condition in developed countries [1]. Approximately 800,000 strokes are reported in the United States every year leading to 200,000 deaths, almost 1 out of every 16 deaths [2, 3]. For those who survive, it is the most common cause of adult disability in the modern world and associated with expensive long-term rehabilitation care [2, 4–6]. Costs are estimated over 60 billion dollars per year in the United States alone [2, 4, 7]. More than 80% of stroke victims suffer from a disease ischemic in nature due to a thrombus or thromboembolism, with the remainder haemorrhagic [2].

During stroke, a core area of tissue dies due to underperfusion and an area of hypoperfused tissue with some collateral vessels remains salvageable (penumbra) if revascularised in a timely manner [8]. The NIHSS (National Institute of Health Stroke Score) is a quick tool to clinically estimate the extent and the severity of it. The score is shown in Table 1.

Urgent reperfusion of the ischaemic brain remains the first target of stroke treatment, either by intravenous medical therapy or by endovascular intervention. Restoration of blood flow to ischaemic tissue has been shown to improve functional outcome and decrease mortality at 3 months compared to no revascularization [9]. Indeed studies have estimated 1.8 days of added healthy life benefit for each minute reduction in time to treatment [10]. An Australian study found that 69% of patients with acute ischaemic stroke were not eligible for thrombolysis due to delay in presentation [11]. Currently intravenous recombinant tissue plasminogen activator (tPA) is only recommended to be used within the 4.5-hour window beyond which the risk of intracranial bleeding may outweigh the benefits [12]. Implementation of stroke systems of care as well as specialised mobile stoke units has proven to reduce the mean time to treatment [13].

Intravenous tPA has been widely accepted as the first-line drug of choice for thrombolysis since FDA approval in 1996 [14]. However, the short therapeutic window, risk of intracranial haemorrhage, and limited efficacy at recanalisation of large vessel occlusion have spurred the development of endovascular treatments as well as other thrombolytic agents which have higher fibrin affinity and better safety profile.

This paper is part of a two-part review on advances in stroke treatment and will focus on medical treatment over the past decade and discuss the implications for future research and treatment.

## 2. Intravenous Thrombolysis

### 2.1. Tissue Plasminogen Activator (tPA)

Despite enormous advancement in the therapeutic options available, the standard treatment for an ischemic stroke is still intravenous thrombolysis following a noncontrast CT brain. Intravenous tPA is administered at a dose of 0.9 mg/kg, with a maximum dose of 90 mg. Ten percent of the medication is given as a bolus and the remainder is infused over 60 minutes [2, 34, 35]. Intravenous tPA therapy in the first 3 hours after stroke onset was demonstrated to be beneficial in the NINDS (National Institute of Neurological Disorders and Stroke) study, which reported a significantly greater proportion of patients having a normal or near normal outcome compared to placebo (38 versus 21 percent, *P* = 0.001) [16].

In 2009, the ECASS 3 study (European Cooperative Acute Stroke Study 3) demonstrated that patients treated with intravenous tPA in the 3–4.5-hour window showed improved outcome compared to placebo (mRS 0-1 in 52 versus 45 percent, *P* = 0.04) with no increase in mortality [17]. This led to the American Heart Association (AHA/ASA) guidelines for intravenous tPA administration to be revised to increase the window of treatment from 3 hours to 4.5 hours given certain limitations and patient-specific criteria (patients with age >80, NIHSS > 25, previous stroke and diabetes, and anticoagulant use were excluded) [1, 34, 36].

The effectiveness of intravenous tPA for use between 4.5 and 6 hours after stroke onset is inconclusive. The results of the IST-3 trial which enrolled 3035 patients within 6 hours of stroke onset showed a greater rate of symptomatic intracranial haemorrhage and mortality, but only insignificant trend towards favourable outcome at 6 months in IV tPA versus control group, 37 versus 35 percent (*P* = 0.181) [18]. The results of the large Ischemic Stroke Recorded in the Safe Implementation (SITS-IST) registry on 29,619 patients did not show worse outcome in patients treated within 4.5 to 6 hours of stroke compared to patients treated within 4.5 or 3 hours [19].

Researchers have also investigated the combination of intravenous tPA and heparin or antiplatelet therapy to prevent reocclusion of vessels. Although not statistically significant, a trend towards more favourable outcome in patients treated with intravenous tPA combined with low molecular weight heparin at presentation was shown. This was associated with a small increased risk of symptomatic intracranial haemorrhage [37, 38]. The Antiplatelet Therapy in Combination with Recombinant tPA Thrombolysis in Ischemic Stroke (ARTIS) study showed that use of 300 mg intravenous acetyl salicylic acid within 1.5 hours of tPA did not improve outcome at 3 months and increased the rate of symptomatic intracranial haemorrhage [20, 37, 39]. Consistent with current guidelines, there is no evidence to support initiation of antiplatelet within the first 24 hours after tPA is administered.

### 2.2. Other Thrombolytic Agents

Conventional thrombolytic agents like alteplase (recombinant tPA) and prourokinase work by converting plasminogen into active plasmin [2, 17, 40, 41]. Although alteplase is the only FDA approved treatment for acute ischemic stroke, newer agents are emerging with the goal to improve the risk-benefit profile of thrombolysis. There are also concerns that alteplase may have negative effects on the ischaemic brain, including cytotoxicity and increased permeability of the blood-brain-barrier (BBB) facilitating cerebral oedema [42]. Efficacy of new agents like tenecteplase, reteplase, plasmin, microplasmin, and desmoteplase and their combination therapy is now being investigated [2, 36, 37, 43].

### 2.3. Tenecteplase

Tenecteplase is a semisynthetic tPA structurally modified to have increased half-life and fibrin affinity and has shown promise in the treatment of ischemic stroke [37, 39, 44]. Parsons et al. (2012) randomised 75 patients to receive alteplase (0.9 mg per kilogram of body weight) or tenecteplase (0.1 mg per kilogram or 0.25 mg per kilogram) and showed that the two tenecteplase groups had greater reperfusion compared to alteplase and the high dose tenecteplase group was superior to the lower dose group in all outcome measures [21].

Smadja et al. (2011) investigated the efficacy of 0.1 mg per kilogram intravenous bolus of tenecteplase as an adjuvant in MCA (M1) occlusions not responsive to intravenous tPA. Out of the 13 patients treated, the recanalisation (TIMI 2/3) rate was 100% and modified Rankin Scale (mRS) 0-1 achieved in 69% of patients at 90 days [45].

Further studies are ongoing, including The Norwegian Tenecteplase Stroke Trial (NOR-TEST) which is a randomised controlled trial comparing efficacy and safety of tenecteplase versus alteplase in stroke patients who present within 4.5 hours of stroke onset as well as comparing them as bridging therapy prior to embolectomy [46].

### 2.4. Desmoteplase

Desmoteplase is a plasminogen activator extracted from the vampire bat saliva, with higher fibrin affinity compared with tPA and long half-life, making it a promising thrombolytic agent. The Desmoteplase in Acute Stroke (DIAS) study investigated the safety and efficacy of various doses of intravenous desmoteplase in patients with perfusion-diffusion mismatch on MRI up to 3 hours from stroke onset. Part 1 of the trial was terminated prematurely due to high rates of symptomatic intracranial haemorrhage (up to 30%) but the second part with lower weight adjusted desmoteplase doses showed increased reperfusion and better functional outcomes in the desmoteplase group compared to placebo [22, 37].

DIAS was followed by the phase II placebo-controlled Dose Escalation of Desmoteplase for Acute Ischemic Stroke (DEDAS) study, which demonstrated safety and efficacy of 125 *μ*g per kilogram dose of intravenous desmoteplase in acute ischemic stroke, although only 25 patients were included in the treatment groups [14, 37].

The subsequent small phase III study, DIAS-2, did not show a benefit of desmoteplase over placebo. This could be explained by a number of factors including the milder stroke severity scores of patients in the study, small core, and mismatch lesion volumes and only 30% of patients in the study had proximal vessel occlusion [4, 37]. Further studies are warranted and phase III DIAS-3 and DIAS-4 trials are now ongoing to evaluate safety and efficacy of 90 ug per kilogram bolus given 3–9 hours after stroke onset [47].

### 2.5. Ancrod

Ancrod is a serine protease, extracted from the venom of the Malayan pit viper that reduces blood fibrinogen levels when injected intravenously. This indirectly leads to anticoagulation, reduced blood viscosity, and increased circulation to affected areas of the brain [48]. It was initially shown to be beneficial in acute ischemic stroke if started within 3 hours of symptom onset [23, 37, 49]. The Stroke Treatment with Ancrod Trial (STAT) randomised 500 patients who presented within 3 hours of stroke onset to receive an infusion of ancrod or placebo over 72 hours and 1-hour infusions at 96 and 120 hours. Better functional outcome was observed in the ancrod group versus placebo, 42.2% versus 34.4%, with a *P* value of 0.04. However the rate of symptomatic intracranial haemorrhage was insignificantly greater in the ancrod group, 5.2% versus 2%, *P* value of 0.06 [23]. Subsequent studies extending the treatment window to 6 hours from onset have not been able to demonstrate any significant difference in clinical outcome [37, 49–51].

### 2.6. Glycoprotein IIb/IIIa Antagonists

Glycoprotein IIb/IIIa inhibitors may prevent platelet activation thus preventing reocclusion as well as facilitating more complete and faster thrombus breakdown [52]. They have been shown as an adjuvant to improve coronary recanalisation in acute myocardial infarction with more TIMI 3 reperfusion in phase II studies but no significant final outcome improvement in the phase III study [37, 53, 54].

Safety of Tirofiban in Acute Ischemic Stroke (SaTIS) is a phase II placebo-controlled study on monotherapy with intravenous tirofiban in patients presenting up to 22 hours after onset. The study confirmed its safety; however there was no neurological/functional benefit found compared with placebo at 5 months except for lower mortality shown in the treatment group [24, 37]. The subsequent Abciximab in Emergency Treatment of Stroke Trial (AbESTT-II), an attempt for a phase III study on GP IIb/IIIa inhibitor monotherapy, was terminated prematurely because of an unfavourable risk-benefit profile. No benefit in neurological recovery was seen in any of the cohorts (within 5-hour onset, between 5-6 hours and wake-up strokes) in the abciximab group compared to placebo. Instead, there was a significant increase in symptomatic intracranial haemorrhage. The authors conclude that intravenous abciximab does not have a role in the management of patients with acute ischaemic stroke [25, 37, 55].

Efficacy and safety of combined intravenous tPA and eptifibatide compared with intravenous tPA alone were investigated in the phase II Combined Approach to Lysis Utilizing Eptifibatide and Recombinant Tissue Plasminogen Activator in Acute Ischemic Stroke—Enhanced Regimen stroke trial (CLEAR-ER) study. The combined treatment group had a lower rate of symptomatic intracranial haemorrhage (2%) and showed a trend towards better functional outcome, with 49.5% achieving mRS 0-1 versus 36% in the standard tPA group [26].

### 2.7. Argatroban

Argatroban is a direct thrombin inhibitor which has demonstrated safety in the Argatroban Anticoagulation in Patients with Acute Ischemic Stroke (ARGIS-I) trial. Patients were randomised to receive a high or low dose intravenous infusion of argatroban or placebo within 12 hours of stroke onset. The rate of symptomatic haemorrhage was nonsignificantly higher in the argatroban groups (5% and 3% versus 0% in placebo); however, the argatroban groups did not show any benefit in functional outcome compared to placebo. Use of argatroban as an adjuvant to intravenous tPA was investigated in the Argatroban TPA Stroke (ARTTS) study and showed 63% complete recanalisation rate at 24 hours [27, 28, 32, 37, 56–59]. Phase II ARTTS-2 (Randomized Controlled Trial of Argatroban with tPA for Acute Stroke) is currently recruiting patients randomised to receive a high or low dose of argatroban infusion for 48 hours and intravenous tPA versus tPA alone and is expected to be completed in 2015 [60].

These important trials have been summarized in Table 2.

## 3. Factors Affecting Outcome of Thrombolysis

As mentioned before, restoration of cerebral blood flow to ischaemic brain tissue with clot lysis and recanalisation is the immediate aim of thrombolytic therapy. A meta-analysis on 2006 patients showed that recanalisation compared to no recanalisation was associated with good functional outcome (OR 4.43, 95% CI 3.32–5.91) and reduced mortality (OR 0.24, 95% CI 0.16–0.35) at 3 months [9]. The outcome of thrombolysis in ischemic stroke depends on multiple factors including thrombus type, location and the extent, collateral circulation, underlying comorbidities, patient's age, time to commencement of the treatment, and time to recanalisation [2].

Studies have shown that large calibre proximal arteries are unlikely to be responsive to chemical intravenous thrombolysis alone [37, 61, 62]. On transcranial ultrasound study, intravenous thrombolysis showed recanalisation rates of 44.2% for distal middle cerebral artery (M2) occlusion, which drops significantly to 30% and 6% for proximal MCA (M1) and distal ICA, respectively [37, 62]. While studies report wide variation in recanalisation rates for large proximal cerebral artery occlusions with intravenous thrombolysis alone, even the highest rates support the fact that there is room for improvement [63].

Clinical trials have also shown that the likelihood of recanalisation negatively correlates with the thrombus burden, with those having a clot less than 8 mm in length having a much better chance to achieve recanalisation [2, 64].

Clot composition may also determine effectiveness of thrombolysis. A study found that fibrin rich cardioembolic thrombus achieved faster and more frequent recanalisation with tPA compared to large vessel atherosclerotic lesions [63]. In a case series with complete or partial MCA recanalisation after intravenous tPA, 20% of patients had early reocclusion and risk factors for this include NIHSS score more than 16 at baseline and severe ipsilateral carotid artery disease, which is defined as more than 70% stenosis in this study [65].

MRI/CT perfusion imaging studies in acute stroke are showing promise to provide a basis to select patients with salvageable brain that will do well past the traditional 3- to 4.5-hour treatment window as well as selection of patients who present with wake-up stroke. This is investigated in the Diffusion and Perfusion Imaging Evaluation for Understanding Stroke Evaluation (DEFUSE) study and MRI Profile and Response to Endovascular Reperfusion after Stroke (DEFUSE-2) study. Both studies showed that perfusion mismatched patients who had early reperfusion after tPA or endovascular stroke treatment had more favourable clinical outcomes and attenuation of infarct growth. The studies also highlighted a subset of patients with a “malignant mismatch” profile characterised by large DWI, more than 100 mL, who were more likely to have serious intracranial haemorrhage and poor outcome after reperfusion [29, 30].

The effects of alteplase beyond 3 hours after stroke in the Echoplanar Imaging Thrombolytic Evaluation Trial (EPITHET) is a randomised controlled trial looking at intravenous tPA versus placebo in patients with a perfusion mismatch 3 to 6 hours after stoke onset. Compared to placebo, intravenous tPA was nonsignificantly associated with better clinical outcome at 90 days but significantly associated with higher rates of reperfusion, 56% versus 26%, with a *P* value of 0.01. The study also found a significant association between overall reperfusion with better clinical outcome and less infarct growth [31]. Further randomised controlled trials are now underway to assess if patients with significant penumbra mismatch at 3 to 9 hours from onset would benefit from tPA (EXTEND study) [66].

WAKE-UP is another ongoing randomised controlled trial that uses DWI-FLAIR mismatch to identify patients for intravenous thrombolysis with tPA amongst patients who wake up with stroke symptoms [67].

## 4. Other Emerging Treatments

### 4.1. Sonothrombolysis

The Combined Lysis Of Thrombus in Brain Ischemia Using Transcranial Ultrasound and Systemic tPA (CLOTBUST) study showed that the thrombolytic efficacy of intravenous tPA was increased, presumably due to the separating effect of energy delivered by sound waves on fibrin strands in the thrombus [32, 37, 68]. Although no significant improvement in clinical outcome was detected (study was not powered for clinical outcome) it was shown that transcranial emission of a 2 MHz sound beam for 2 hours, targeted towards the thrombus and proximal MCA, significantly increased the rate of complete recanalisation with tPA compared with placebo (38% versus 13%) with no increase in complications or risk of haemorrhage [32, 37, 68]. Studies using lower frequency beams for better mechanical advantage were shown to be unsafe [37, 69].

Phase III randomised study CLOTBUST-ER is ongoing and evaluates the efficacy and safety of the Clotbust-ER ultrasound (ultrasonic headframe) device when used in combination with standard intravenous tPA. The study has completed enrolment of half the subjects (460 of 800) without being halted on interim analysis. Primary efficacy endpoint is 90-day functional recovery and incidence of symptomatic intracranial haemorrhage (sICH) as the primary safety endpoint [70].

Ultrasound contrast agents have showed that further energy can be delivered to the tissue when oscillating microbubbles cavitate, facilitating thrombus degradation and likely promoting recanalization [37]. The Transcranial Ultra-Sound in Clinical SoNo-Thrombolysis (TUSCAN) study looked into the safety and efficacy of ultrasound assisted tPA thrombolysis, using escalating doses of lipid-based microbubbles (Microsphere), which are resistant to transpulmonary passage. The study demonstrated 67% complete recanalisation following the first dose [33, 37]. However, the study was terminated prematurely due to significantly increased risk of intracranial haemorrhage after the second dose [33, 37].

In another study, three different groups of stroke patients within 3 hours received tPA alone, tPA and ultrasound, and combined tPA, ultrasound, and microbubbles. The final comparison demonstrated safety of the microbubbles with no increased treatment complications and significantly higher recanalisation rate [37, 71], warranting further comprehensive studies into efficacy of sonothrombolysis [17, 69].

## 5. Conclusion

This paper highlights the evidence for intravenous tPA thrombolysis and newer adjuvant therapies. The common goal in chemical or mechanical recanalisation is to establish flow to the ischaemic areas of the brain. There is clearly a role for thrombolysis in the acute management of stroke care since it is easily accessible in primary stroke centres and does not require a catheter lab.

There is an ongoing search for thrombolytic agents that are more effective than tPA and can be used safely over a longer period to maximise the benefit to a greater number of patients. Tenecteplase is one such agent that has shown promising results. Adjuvant antithrombotics such as argatroban have shown high recanalisation rates with intravenous tPA in a single arm study and await confirmation of superiority in an ongoing randomised trial. Adjuvant use of transcranial ultrasound has been shown to improve recanalisation rates with intravenous tPA in phase II trials and is now undergoing phase III testing. The use of neuroimaging to provide more robust selection criteria to delineating patients with perfusion mismatch is a key focus of research, with ongoing phase III trials.

## Figures and Tables

**Table 1:** National Institutes of Health Stroke Scale.

Tested item	Title	Response and score
1A	Level of consciousness	0 = alert
		1 = drowsy
		2 = obtunded
		3 = coma/unresponsive

1B	Orientation questions (2)	0 = answers both correctly
		1 = answers one correctly
		2 = answers neither correctly

1C	Response to commands (2)	0 = performs both tasks correctly
		1 = performs one task correctly
		2 = performs neither

2	Gaze	0 = normal horizontal movements
		1 = partial gaze palsy
		2 = complete gaze palsy

3	Visual fields	0 = no visual field defect
		1 = partial hemianopia
		2 = complete hemianopia
		3 = bilateral hemianopia

4	Facial movement	0 = normal
		1 = minor facial weakness
		2 = partial facial weakness
		3 = complete unilateral palsy

5	Motor function (arm) (a) Left (b) Right	0 = no drift
		1 = drift before 5 seconds
		2 = falls before 10 seconds
		3 = no effort against gravity
		4 = no movement

6	Motor function (leg) (a) Left (b) Right	0 = no drift
		1 = drift before 5 seconds
		2 = falls before 5 seconds
		3 = no effort against gravity
		4 = no movement

7	Limb ataxia	0 = no ataxia
		1 = ataxia in 1 limb
		2 = ataxia in 2 limbs

8	Sensory	0 = no sensory loss
		1 = mild sensory loss
		2 = severe sensory loss

9	Language	0 = normal
		1 = mild aphasia
		2 = severe aphasia
		3 = mute or global aphasia

		0 = normal
10	Articulation	1 = mild dysarthria
		2 = severe dysarthria

11	Extinction or inattention	0 = absent
		1 = mild (loss 1 sensory modality)
		2 = severe (loss 2 modalities)

Adapted from Adams et al. (2007) [15].

**Table 2:** Summary of articles.

Study	Design	Patient groups^*^	Baseline NIHSS	Time from onset	Time to treatment	Symptomatic ICH%	Mortality% (90-day)	Functional outcome at 3 months
Tissue plasminogen activator (tPA)								

NINDS: parts 1 and 2 (1995) [16]	RCT	*N* = 624 Part 1: improvement >4 in NIHSS within 24 hours Part 2: clinical outcome at 3 months G1: IV tPA (0.9 mg/kg, max 90 mg) G2: placebo	Median (range) Part 1: G1: 14 (1–37) G2: 14 (1–32) Part 2: G1: 14 (2–37) G2: 15 (2–33)	Within 3 hours	0–90 mins G1: 50% G2: 46% 91–180 mins G1: 50% G2: 54%	Within 36 hours: G1: 6.4% G2: 0.6% (*P* < 0.001)	G1: 17% G2: 21% (*P* = 0.30)	mRS 0-1 0–90 mins G1: 40% G2: 28% (*P* = 0.035) 91–180 mins G1: 45% G2: 25% (*P* < 0.001)

ECASS 3 (2009) [17]	RCT	*N* = 821 G1: IV-tPA (0.9 mg/kg, max 90 mg) G2: placebo	Mean (SD) G1: 10.7 (5.6) G2: 11.6 (5.9)	Between 3 and 4.5 hours	Mean mins G1: 239 G2: 238	G1: 2.4% G2: 0.2% (*P* = 0.008)	G1: 7.7% G2: 8.4% (*P* = 0.68)	mRS 0-1 G1: 52.4% G2: 45.2% (*P* = 0.04)

IST-3 (2012) [18]	RCT	*N* = 3035 G1: IV tPA G2: control	G1: 0–5 20% 6–10 28% 11–15 20% 16–20 18% >20 14% G2: 0–5 20% 6–10 28% 11–15 19% 16–20 18% >20 14%	Within 6 hours	Mean mins G1: 252 (72) G2: —	Within 7 days G1: 7% G2: 1% (*P*≤ 0.0001)	Within 7 days G1: 11% G2: 7%, *P* = 0.001 Between 7 days and 6 months: G1: 16% G2: 20%, (*P* = 0.002)	Independent patients (Oxford Handicap score of 0–6) and alive at 6 months: G1: 37% G2: 35% (*P* = 0.181)

SITS-ISTR (2013) [19]	Observational registry	*N* = 29618 IV tPA in different treatment times G1: 4.5–6 hours G2: 3–4.5 hours G3: within 3 hours	Median (IQR) G1: 9 (6–15) G2: 9 (6–15) G3: 12 (7–17)	Within 3, 4.5, or 6 hours	Median (IQR) mins G1: 295 (289–315) G2: 210 (195–235) G3: 138 (112–162)	G1: 2.6% (*P* = 0.17) G2: 1.8% (*P* = 0.27) G3: 1.5%	G1: 11.8% (*P* = 0.99) G2: 11.1% (*P* = 0.21) G3: 11.8%	mRS 0–2 G1: 61.3% (*P* = 0.4) G2: 62.7% (*P* < 0.01) G3: 58.4%

ARTIS (2012) [20]	RCT	*N* = 642, G1: 300 mg IV aspirin 90 mins after IV tPA G2: standard treatment	Median (IQR) G1: 9 (5–12) G2: 9 (5–14)	Within 4.5 hours	Mean (SD) mins G1: 124.4 (54) G2: 127.9 (57.6)	G1: 7.4% G2: 0.7% (*P* = 0.006)	G1: 11.2% G2: 9.7% (*P* = 0.54)	mRS 0–2 G1: 54% G2: 57.2% (*P* = 0.42)

Other thrombolytic agents								

Parsons et al. (2012) [21]	RCT	*N* = 75 G1: alteplase (0.9 mg/kg) G2: tenecteplase (0.1 mg/kg) G3: tenecteplase (0.25 mg/kg)	G1: 14 (2.3) G2: 14.5 (2.3) G3: 14.6 (2.3)	Within 6 hours	Mean (SD) hours G1: 2.7 (0.8) G2: 3.1 (0.9) G3: 3 (0.7)	G1: 12% G2+3: 4% (*P* = 0.33)	G1: 12% G2+3: 8% (*P* = 0.68)	mRS 0–2 G1: 44% G2+3: 72% (*P* = 0.02) Reperfusion on MRI at 24 hr G1: 55.4% (38.7) G2+3: 79.3% (28.8) (*P* = 0.004) Complete recanalisation at 24 hr G1: 36% G2+3: 58% (*P* = 0.09)

DIAS (2005) [22]	RCT	*N* = 104 patients with perfusion/diffusion mismatch on MRI treated with IV desmoteplase or placebo Part 1 (*N* = 47) trial terminated prematurely due to high rates of sICH (23.5%–30.8%). Part 2 (*N* = 57) had lower weight adjusted doses G1: 62.5 ug/kg G2: 90 ug/kg G3: 125 ug/kg G4: placebo	G1: 13 G2: 12 G3: 12 G4: 8	Between 3 and 9 hours	Mean mins G1: 360 G2: 400 G3: 295 G4: 340	Within 72 hr G1: 0% G2: 6.7% G3: 0% G4: 0%	G1+2+3: 4.4% G4: 9.1%	Favourable clinical outcome (composite score) at 90 days G1: 13.3% (*P* = 0.757) G2: 46.7% (*P* = 0.054) G3: 60% (*P* = 0.009) G4: 18.2% Early reperfusion at 4- to 8-hour (reduction ≥30% mean transit time or ≥2 point improvement in TIMI) G1: 23.1% (*P* = 0.390) G2: 46.7% (*P* = 0.035) G3: 71.4% (*P* = 0.0012) G4: 20% Favourable clinical outcome associated with reperfusion, 53% versus 25% (*P* = 0.0028)

DEDAS (2006) [14]	RCT	*N* = 37 patients with perfusion/diffusion mismatch on MRI treated with IV desmoteplase or placebo G1: 90 ug/kg G2: 125 ug/kg G3: placebo	Median (range) G1: 10 (4–18) G2: 9 (5–19) G3: 12 (6–18)	Between 3 and 9 hours	Median (range) mins G1: 477 (393–568) G2: 420 (222–531) G3: 443 (220–516)	G1: 0% G2: 0% G3: 0%	G1: 7.1% G2: 6.7% G3: 12.5%	Favourable clinical outcome (composite score) G1: 37.5% (*P* = 0.20) G2: 72.7% (*P* = 0.022) G3: 16.7% Early reperfusion at 4- to 8-hour (reduction ≥30% mean transit time or ≥2 point improvement in TIMI) G1: 16.7% (*P* = 0.74) G2: 63.6% (*P* = 0.12) G3: 33.3%

DIAS II (2009) [4]	RCT	*N* = 193 patients diffusion mismatch on MRI treated with IV desmoteplase or placebo G1: 90 ug/kg G2: 125 ug/kg G3: placebo	Median (IQR) G1: 9 (7–14) G2: 9 (7–15) G3: 9 (6–14)	Between 3 and 9 hours	Mean (SD) mins G1: 388 (88) G2: 402 (88) G3: 391 (92)	G1: 3.5% G2: 4.5% G3: 0%	G1: 11% G2: 21% G3: 6%	Favourable clinical outcome (composite score) G1: 47% G2: 36% G3: 46% (*P* = 0.47)

STAT (2000) [23]	RCT	*N* = 500 G1: ancrod 0.167 IU/kg iv per hour for over 72 hours, followed by 0.125 and 0.082 IU/kg over 1 hour at 96 and 120 hours depending on plasma fibrinogen level (target 1.18 to 2.03 micromole/L) G2: placebo	Mean (SD) G1: 23.8 (10) G2: 24.4 (11)	Within 3 hours	Mean (SD) hours G1: 2.7 (0.4) G2: 2.7 (0.5)	G1: 5.2% G2: 2% (*P* = 0.06)	G1: 25.4% G2: 23% (*P* = 0.62)	Favourable outcome (Barthel Index >95) G1: 41.1% G2: 35.3% (*P* = 0.04)

SaTIS (2011) [24]	RCT	*N* = 260 G1: tirofiban infusion (0.4 ug/kg/min over 30 mins + continuous infusion 0.1 ug/kg/min for 48 hr) G2: placebo	Median (range) G1: 6 (4–18) G2: 6 (4–18)	Between 3 and 22 hours	Median hours G1: 9.25 G2: 10.7	Haemorrhagic transformation/parenchymal haemorrhage within 7 days G1: 30% G2: 26.6% (*P* = 0.665)	Mortality at 5 months: G1: 2% G2: 8% (*P* = 0.03)	Mean mRS after 5 months: G1: 1.9 G2: 2.2 (*P* = 0.3)

AbESTT-II (2008) [25]	RCT	*N* = 808 trial terminated prematurely due to unfavourable risk-benefit profile Primary cohort G1: abciximab G2: placebo 5-6 hr cohort G3: abciximab G4: placebo Wake-up cohort G5: abciximab G6: placebo	Mean (SD) G1: 9.9 (5.2) G2: 9.6 (5) G3: 9.5 (4.8) G4: 9.4 (4.9) G5: 10.4 (5) G6: 10 (5)	Primary cohort: within 5 hours 5-6 hr cohort Wake-up cohort: within 3 hours of awakening	Mean hours G1: 3.6 G2: 3.8 G3: 5 G4: 5 G5: 10.8 G6: 12.1	Within 5 days/discharge G1: 5.5% G2: 0.5% (*P* = 0.002) G3: 1.9% G4: 0.6% (*P* = 0.309) G5: 13.6% G6: 5% (*P* = 0.347)	G1: 15.8% G2: 11.5% (*P* = 0.167) G3: 11.3% G4: 10.7% (*P* = 0.887) G5: 27.3% G6: 14.3% (*P* = 0.298)	mRS 0-1 G1: 42.5% G2: 44.5% (*P* = 0.679) G3: 36.3% G4: 40.3% (*P* = 0.462) G5: 9.1% G6: 28.6% (*P* = 0.101)

CLEAR-ER (2013) [26]	RCT	*N* = 126 G1: combination IV 0.6 mg/kg tPA + eptifibatide (135 mcg/kg bolus + 0.75 mcg/kg/min infusion for 2 hr) G2: standard IV tPA	Median (IQR) G1: 12 (9–20) G2: 17 (11–22)	Within 3 hours	Median (IQR) mins G1: 113 (99–135) G2: 129 (90–141)	Within 36 hours G1: 2% G2: 12% (*P* = 0.053)	G1: 19.8% G2: 4% (*P* = 0.78)	mRS 0-1 or return to baseline mRS G1: 49.5% G2: 36% (*P* = 0.23)

ARGIS-1 (2004) [27]	RCT	*N* = 171 G1: IV argatroban (100 ug/kg bolus) + infusion 3 ug/kg/min PT time × 2.5 G2: IV argatroban (100 ug/kg bolus) + infusion 1 ug/kg/min PT time × 1.75 G3: placebo	Median (IQR) G1: 9 (5–22) G2: 10 (5–22) G3: 9 (5–20)	Within 12 hours	N/A	G1: 5.1% (*P* = 0.18) G2: 3.4% (*P* = 0.5) G3: 0%	G1: 11.1% G2: 19.3% G3: 9.3%	mRS 0–2 G1: 51% (*P* = 0.58) G2: 45% (*P* = 0.61) G3: 54%

ARTTS (2012) [28]	Single arm prospective trial	*N* = 65 During standard IV tPA, IV argatroban given (100 ug/kg bolus) + infusion for 2 days with target PT time × 1.75	Median (range) 13 (3–25)	Within 4.5 hours	Median (IQR) mins 128 (94–170)	4.6%	7-day mortality 10.8%	7-day mRS 0-1 29% Complete recanalisation (TIMI 3) at 24 hr 63%

Penumbra neuroimaging								

DEFUSE 2 (2012) [29]	Prospective cohort study	*N* = 138 (98 had endovascular treatment; 104 had MRI profile) G1: *N* = 78 had target mismatch G2: *N* = 26 had no target mismatch	Median (IQR) G1: 14 (11–20) G2: 18 (12–19)	Within 12 hours	Median (IQR) hours G1: 6.2 (4.9–8.1) G2: 3.6 (2.5–4.9)	G1: 7% G2: 19% (*P* = 0.15)	N/A	mRS 0–2 G1 Reperfused 57% No reperfusion 31% (*P* = 0.04) G2 Reperfused 25% No reperfusion 22% (*P* = 1.0) Median (core) lesion growth (IQR) mL G1 Reperfused 30 (5–67) No reperfusion 73 (32–127) (*P* = 0.005) G2 Reperfused 96 (68−159) No reperfusion 34 (4–147) (*P* = 0.21)

DEFUSE (2006) [30]	Prospective cohort study	*N* = 74 IV TPA 0.9 mg/kg G1: target mismatch G2: no target mismatch	Median (IQR) 11.5 (8)	Between 3 and 6 hours	Median (IQR) mins 328 (40)	G1 Reperfused 22% No reperfusion 6.3% G2 Reperfused 25% No reperfusion 0% Malignant profile 50%	N/A	mRS 0–2 G1 Reperfused 50% No reperfusion 13% G2 Reperfused 25% No reperfusion 71% Malignant profile: 17% Mismatch with early reperfusion OR 5.4 (CI 1.1–25.8) for favourable clinical response (*P* = 0.039)

EPITHET (2008) [31]	RCT	*N* = 101 G1: alteplase G2: placebo 86% of patients had perfusion mismatch	Median (range) G1: 14 (4–26) G2: 10 (5–25)	Between 3 and 6 hours	Mean (SD) mins G1: 297 (42) G2: 294 (50)	Within 36 hours G1: 7.7% G2: 0%	G1: 25% G2: 14% (*P* = 0.161)	mRS 0-1 G1: 35% G2: 24% (*P* = 0.153) Reperfusion (>90% reduction between baseline and day 3 PWI volume) G1: 56% G2: 26% (*P* = 0.01) Infarct growth in geometric mean at 90 days G1: 1.24 G2: 1.78 (*P* = 0.239)

Adjuvant/experimental treatments								

CLOTBUST: phase 2 (2004) [32]	RCT	*N* = 126 patients Proximal arterial occlusion on TCD G1: IV 0.9 mg/kg TPA and 2 MHz TCD with max 750 mW power for 2 hours at the target lesion G2: placebo	Median G1: 16 G2: 17	Within 3 hours	Median mins G1: 150 G2: 130	Within 72 hours G1: 4.8% G2: 4.8%	G1: 15% G2: 18% (*P* = 0.4)	mRS 0-1 G1: 42% G2: 29% (*P* = 0.2) Complete recanalisation (TIMI 3) at 2 hours G1: 29% G2: 11% (*P* < 0.001)

TUCSON (2009) [33]	RCT	*N* = 35 Receiving IV tPA with proximal intracranial occlusion G1: microS (MRX-801) infusion over 90 minutes (1.4 mL) G2: microS (MRX-801) infusion over 90 minutes (2.8 mL) G3: control	Median (IQR) G1: 10 (4–14) G2: 16 (9–21) G3: 25 (7–14)	Within 3 hours	Mean (SD) mins G1: 139 (33) G2: 130 (32) G3: 126 (46)	G1: 0% G2: 27% G3: 0% (*P* = 0.028)	G1: 0% G2: 30% G3: 0% (*P* = 0.022)	mRS 0-1 G1: 75% G2: 50% G3: 36% (*P* = 0.167) Complete recanalisation G1: 67% G2: 45% G3: 33% (*P* = 0.255)

^*^G = study groups.
